# Of the Peculiarities of Structure in Individual Parts and Organs and Particular Regions of the Body in the Tuberculous Predisposition

**Published:** 1850-09

**Authors:** Henry Ancele


### PATHOLOGY AND PRACTICE OF MEDICINE.

*Of the peculiarities of Structure in Individual Parts and Organs and particular regions of the Body in the Tuberculous Predisposition.* By Henry Ancell, Esq.,—The morbid state of the blood and blastema, and the malnutrition of cells and fibres are not only exhibited in the various tissues, but some remarkable peculiarities are observed in the form and development and the general characteristics of individual parts made up of different tissues. Generally there is a defect of symmetry, with a tendency, according to Hufeland and others, to the disunion of various parts of the body at the median line. The median line is also frequently removed from the centre, the lateral halves of the body being unequal: a confirmation, it is remarked by Lugol, of the worst augury, as respects future disease. Many of the features of the face present highly characteristic appearances. In some of the most unequivocal cases the structure of the eyes exhibit several peculiarities. A bluish tint of the sclerotic coat, even in adult age, resembling that presented in the earliest infancy, is very common ; countenancing the idea, already several times adverted to, of an arrest of development. This coat is sometimes pearl-white, which, according to Hufeland, betrays a tendency to the mesenteric variety of the disease ; it is also frequently traversed by streaks of enlarged and injected blood-vessels. The eyes, at other times, particularly in young children, are haggard and inexpressive, and the Meibomian glands are liable to be spuriously hypertrophied. The eyelids are sometimes thick or œdematous. The mouth is unsymmetrical—small or large, or too much arched ; the upper or lower lip, or both lips, are liable to become tumid from a spurious hypertrophy, consisting of an infiltration of the mucous, cutaneous, and cellular tissues of which they are composed; and as stated of the cellular tissue itself, and from a similar cause, this tumidity is subject to a rapid increase or decrease from slight variations of the general health, and is frequently more perceptible in the morning than during the remainder of the day. In the upper lip, the swelling frequently extends to the columnæ nasi and lower part of the nostrils, and there is occasionally a chap in the middle of the lip. The nose is sometimes swollen, red, and shining, and the alæ nasi spread in a remarkable manner. The lobes of the ears are also sometimes affected with a tumidity similar in its nature to that of the lips. These peculiarities, with the modified conditions of the dermoid, cellular, and muscular structures incidental to the tuberculous predisposition, according to their degree and varieties, and the mode in which they are combined, give the countenance several well-marked aspects. There is the pale, pasty, full cheek, with large upper lip and wide spread alæ nasi of early childhood; the unnaturally fair or white complexion, resembling blanched wax, with large and conspicuous veins, combined with the roseate hue already described; the full dark eye, with dilated pupil, long eyelashes, regular features, placid expression, but extremely delicate skin; or similar features, with equally fine skin, and a fair florid complexion. There are combinations, sometimes of great beauty, sometimes the contrary, among the associations, which, with other peculiarities belonging to the tuberculous habit, strike the experienced medical observer, and frequently lead him at once to the inference, that the structures have been developed from a defective blastema, and that probably they continue to be nourished from vitiated blood endowed with a low degree of vitality. The peculiarities of tint in different parts of the body, as in the skin, hair, and capillaries, depend in part upon the tenuity and transparency of the tubes and vessels themselves, which traverse their structures, allowing the fluids, whether circulating or stagnant, to reflect the rays of light through them, and in part upon a change of tint of the parenchyma, the whole of the phenomena being ultimately referrible to the blood; which is not only, as we have seen, deficient in red corpuscles, but in which the yellow and blue coloring principles of the liquor sanguinis have probably undergone some unknown changes, either in quantity or quality, involving modifications of tint in the blastema of the different organic tissues.

In the tuberculous predisposition the lymphatic glands over the whole body, but most especially in the neck, are believed to be larger than usual, although no accurate observations have been made on this point. They are unquestionably, very prone to become enlarged, and in that state are regarded as one of the most certain diagnostic signs of that important variety of tuberculosis which comes under the designation of scrofula. The same remark applies to the tonsils, which, without any apparent deviation from the usual state of health, are liable to become spuriously hypertrophied, sometimes to the extent of affecting the voice and respiration. Baumes refers the habitual enlargement of the glands to an increased quantity of the blastema or nutritive fluid of the cellular tissue of these organs, which fluid he believes to be of an acescent quality. We have given the grounds upon which the acid theory, of tuberculous affections generally, rested, and there are no direct experiments to prove this acidity in the glands. The abdomen, particularly in tuberculous infants and children, is frequently very much enlarged; a circumstance readily explained by the weak organization of the intestinal tunics and of the abdominal parietes, giving rise to habitual over-distension from flatulence and other dyspeptic effects produced by slight causes, and in some cases this distension is increased by enlargement of the mesenteric glands, which are liable to changes analogous to those of the external glands.

As respects the viscera, great doubt hangs over the real condition of their organization in the tuberculous predisposition. Pathologists have made numerous observations of the morbid appearances exhibited after death from the different varieties of tuberculosis; but in almost all these cases some local disease has for a long time existed, and contributes to destroy the patient. These pathological appearances will have to be considered under the head of tuberculosis in a state of actual disease; but it is difficult, if not impossible, to draw any inference from them as to the state of the same organs, where only the predisposition to disease exists. No doubt they participate in the general malnutrition of the elementary tissues of which they are composed, and the defective cell-growth constituting the malnutrition, with the deteriorated qualities of the blood which pervades the organs, occasion the functional derangement about to be described. Most likely this constitutes the whole of their pathology in the tuberculous predisposition;—an inference supported by the fact, that where tuberculous subjects have died of diseases other than tubercular disease, no constant pathological appearances are recorded.

Before terminating this account of the structural peculiarities of the tuberculous predisposition, the condition of the heart demands special consideration. It is very generally admitted that, in some tuberculous diseases at least, the heart is within its usual dimensions, and this would be consistent with the malnutrition of its cellular and fibrous structures and corresponding with malnutrition of these tissues generally. According to Bigot, of Geneva, the linear dimensions of the heart, are *cœteris paribus*, in every way less in phthisis than under ordinary circumstances. Louis observed in 112 cases, in which death was caused by phthisis, that the heart, in the majority, was small, and very frequently not more than one-half or two-thirds its ordinary size. Dr. Clark states that he regards a small heart as a strong "predisposing cause" of consumption, and Mr. F. A. Bulley, and I believe other writers in our own country, have insisted upon this point. This, it is necessary, however, to state, has been disputed by others. Andral appears to have found the heart larger than nature in a majority of phthisical subjects, and the late Dr. Clendenning arrived at a somewhat similar conclusion; he found that the heart weighed considerably more in phthisical subjects than in health, but in chronic diseases generally there was an increase of weight in the viscera; and the increase or weight in the heart in phthisis, relative to the increase in weight of the other viscera, was very small indeed. New observations are required as to the size, density, and general nutritive condition of the heart in tuberculosis, distinguishing as accurately as possible its state of nutrition in the earlier and later stages of the affection, in the general disease, and after local affections of the viscera, particularly of the lungs, have for a long time existed, and previous and subsequent to the supervention of the hectic fever. I have been in the habit, for many years past, of examining the heart in fatal cases of phthisis, and to the sight and touch it has almost invariably appeared to me to be of small dimensions or defective substance. The physical signs derived from percussion of the chest have also, in the main, favored this view. In the minor degrees of the tuberculous predisposition, in which the cellular structures alone appear to have been imperfectly developed, leaving the fibrous structures comparatively intact the heart may not only be as large as natural, in relation to the organization of the individual, but even larger; still I consider that in the more decided cases the heart is small and its nutrition imperfect, and that this condition of the organ is in relation to the low vitality of cells, cytoblastema, and blood; and from the large amount of fibrous, as compared with cellular structure, the malnutrition of this organ may be very unequal in different cases.—*London Med. Times.*

